# Draft Genome Sequence of *Natronolimnobius baerhuensis* CGMCC 1.3597^T^, an Aerobic Haloalkaliphilic Archaeon Isolated from a Soda Lake

**DOI:** 10.1128/genomeA.00710-17

**Published:** 2017-09-21

**Authors:** Xiaomeng Guo, Ziya Liao, Yanchun Yan, Mark Holtzapple, Qingping Hu, Baisuo Zhao

**Affiliations:** a College of Life Science, Shanxi Normal University, Linfen, Shanxi, People’s Republic of China; b Graduate School, Chinese Academy of Agricultural Sciences, Beijing, People’s Republic of China; c Department of Chemical Engineering, Texas A&M University, College Station, Texas, USA

## Abstract

The haloalkaliphilic archaeon *Natronolimnobius baerhuensis* was isolated from a soda lake in Inner Mongolia (China), growing optimally at about 20% NaCl and pH 9.0. The draft genome consists of approximately 3.91 Mb and contains 3,810 predicted genes. Some genes that regulate intracellular osmotic stress and pH homeostasis were identified, providing insight into specific adaptations to this double-extreme environment.

## GENOME ANNOUNCEMENT

The mesophilic haloalkaliphile *Natronolimnobius baerhuensis* (strain CGMCC 1.3597^T^ = JCM 12253^T^) is a strictly aerobic Gram-negative archaeon isolated from soda lakes in Inner Mongolia, China (1). This strain is the type species of the genus *Natronolimnobius*, which thus far contains only two haloalkaliphiles. *N. baerhuensis* is an extremely halophilic alkaliphile because it requires at least 15% NaCl (optimum, 20% NaCl) and grows at pH 7.0 to 10.0 (optimum, pH 9.0). To gain insight into the survival and adaptation strategies under double-extreme environmental stress, the draft whole genome of *N. baerhuensis* strain CGMCC 1.3597^T^ was sequenced.

Genomic DNA was extracted using a microbial DNA isolation kit according to the manufacturer’s instructions (New Industry, Beijing, China). Library construction for genome sequencing was prepared using the NEBNext Ultra DNA library prep kit of Illumina (2). Sequencing was performed by the Illumina HiSeq 4000 sequencer with a paired-end read length of 2 × 150 bp at approximately 200× coverage. Genomic contigs were *de novo* assembled using MicrobeTrakr plus version 0.9.1 (incorporates Velvet version 1.2.09). Quake (3) and BWA (4) were used in preassembly and postassembly sequence correction, respectively. A total of 5,752,927 reads were assembled into eight contigs, with a total length of 3,914,301 bp, G+C content of 59.2%, and *N*_50_ value of 1,261,254 bp. The assembled genome was annotated using NCBI Prokaryotic Genome Annotation Pipeline (http://www.ncbi.nlm.nih.gov/genome/annotation_prok) (5) and checked by the GenBank curation team. Among the 3,810 genes predicted, 3,662 were potential protein-coding genes (CDSs). Also identified were 45 RNAs, including 8 rRNAs (six 5S RNAs, one 16S RNA, and one 23S RNA), 45 tRNAs, and two noncoding RNAs (ncRNAs).

Genome sequence analysis revealed various genes that code for proteins potentially related to the adaptation of *N. baerhuensis* to highly saline and alkaline environments. Two genes encode trehalose-6-phosphate synthase (TPS) and trehalose-6-phosphate phosphatase (TPP) for trehalose biosynthesis, and two genes encode sodium-solute symporter (SSS). These genes implied that *N. baerhuensis* is involved in the maintenance of osmotic balance via the compatible-solutes strategy under hypersaline conditions (6, 7). The analysis identified 10 genes (eight TrkA-type and two TrkH-type) that are responsible for K^+^ uptake systems, which indicates that *N. baerhuensis* also achieves an isosmotic cytoplasm using K^+^ as an osmolyte via the salt-in strategy (6, 7). *N. baerhuensis* is an obligate halophilic alkaliphile and therefore must have mechanisms for cytoplasmic acidification because it has 4 genes of the multisubunit Na^+^/H^+^ antiporter (CPA-3 family), 4 genes of the monovalent cation/H^+^ antiporters (CPA-1 family), and 1 gene of the Na^+^/H^+^ antiporter (NhaC type). Furthermore, five genes were identified from the Kef-type K^+^ efflux system, which protects cells against the detrimental effects of electrophilic compounds via cytoplasmic acidification. In this report, the predicted genes play essential roles in maintaining stable osmotic balance and pH homeostasis to avoid cell intoxication.

### Accession number(s).

The draft genome assembly of *N. baerhuensis* CGMCC 1.3597^T^ has been deposited at DDBJ/ENA/GenBank under the accession number MWPH00000000.
